# Myocardial Priority Promotes Cardiovascular Recovery for Acute Type A Aortic Dissection Combined with Coronary Artery Disease Undergoing Aortic Arch Surgery

**DOI:** 10.3390/jpm13091296

**Published:** 2023-08-25

**Authors:** Lian Duan, Chengliang Zhang, Xuliang Chen, E Wang, Zhi Ye, Yanying Duan, Lingjin Huang

**Affiliations:** 1 Department of Cardiovascular Surgery, National Clinical Research Center for Geriatric Disorders, Xiangya Hospital, Central South University, Changsha 410008, China; 2 Department of Anesthesiology, National Clinical Research Center for Geriatric Disorders, Xiangya Hospital, Central South University, Changsha 410008, China; ewang324@csu.edu.cn (E.W.);; 3 Department of Occupational and Environmental Health, Public Health School, Central South University, Changsha 410008, China

**Keywords:** type A dissection, coronary artery stenosis, aortic arch surgery, myocardial protection, blood conservation, branch priority

## Abstract

The optimal surgical strategy for acute type A aortic dissection (ATAAD) with coronary artery disease (CAD) remains unclear. The goal of this study was to investigate the cardiovascular protective effects of the myocardial priority (MP) strategy or traditional selective cerebral perfusion (SCP) in ATAAD with CAD. A total of 214 adults were analyzed retrospectively, of which 80 underwent the MP strategy intraoperatively. Seventy-nine pairs were propensity-score-matched and divided into SCP and MP groups. The follow-up period ranged from 6 to 36 months. The MP group had a significantly shorter myocardial ischemic time, higher perfusion flow, higher radial artery pressure, and lower incidence of NIRS decrease >20% of the base value, but a longer lower limb circulatory arrest and bypass time than the SCP group. Although similar adverse cardiac and cerebrovascular events were observed in both groups, a shorter posthospital stay, less blood loss and transfusion, higher postoperative hemoglobin, lower creatinine, and higher PaO_2_/FiO_2_ were observed in the MP group. Subgroup analysis showed that when the TIMI Risk Score was <4, the MP group had a lower incidence of low cardiac output and lower postoperative cTnI level. The follow-up patients had similar morbidities between the two groups. The novel MP strategy is associated with a shortened myocardial ischemic time, better maintained perfusion of vital organs, and postoperative recovery after surgery for ATAAD combined with non-severe CAD.

## 1. Introduction

When acute type A aortic dissection (ATAAD) and coronary artery disease (CAD) are combined, treatment becomes more challenging [1]. ATAAD requires patients to have effective analgesia and sedation during the perioperative period and to take controlled hypotension measures [2]. Because of coronary artery stenosis, CAD requires that the decrease in arterial blood pressure should not exceed the basic value too much to maintain the minimum value required for the perfusion of vital organs, such as the heart, brain, kidney, and spinal cord [3]. However, some of these patients may have been taking antiplatelet medication before the onset of ATAAD, which increases the risk of bleeding during surgery [4]. 

Anterograde selective cerebral perfusion (SCP) [5], which is the main form of brain protection during hypothermic lower body circulation arrest (LCA) in the surgical treatment of ATAAD involving the aortic arch, can provide physical blood flow but increases the need for axillary incision and cannulation procedures [6]. 

The combination of SCP with myocardial perfusion in pediatric arch surgeries has been reported [7,8] and has reduced cardiac morbidities [9]. Researchers call it cerebral myocardial perfusion or “beating heart priority” [10]. However, early adult cerebral myocardial perfusion aortic procedures are complex and cause unreduced cardiac morbidity [10]. 

Since the beginning of 2017, we modified our myocardial priority (MP) strategy in adult ATAAD surgery combined with CAD and explored whether it could shorten the posthospital stay, or decrease adverse cardiac or cerebrovascular events (ACCE) when compared with SCP operative procedures.

## 2. Materials and Methods

**Figure 1:**
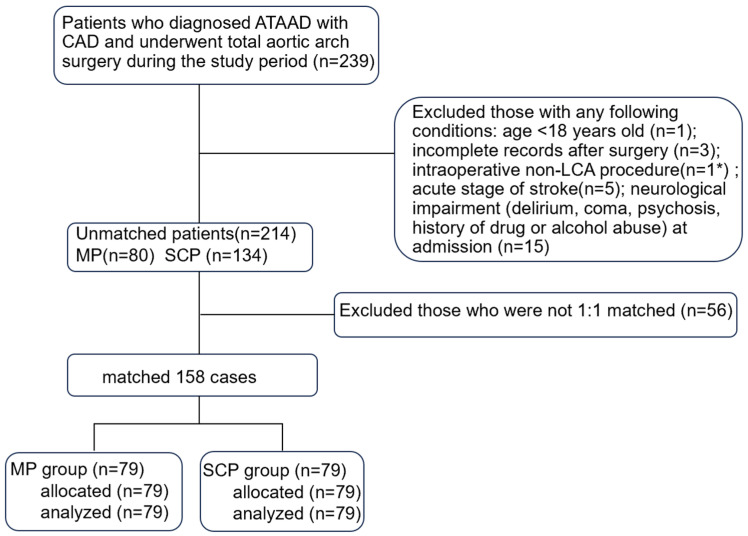
Flow chart of this study. ATAAD, acute type A aortic dissection; CAD, coronary artery disease; LCA, lower limb circulation arrest; MP, myocardial priority; SCP, selective cerebral perfusion. * The patient underwent retrograde inferior vena cava perfusion.

The medical records of 214 consecutive adult patients who were diagnosed with ATAAD and had a history of CAD at the Xiangya Hospital of Central South University from January 2017 to June 2020 were analyzed retrospectively. CAD was diagnosed in a medical institution before or at the same time as the ATAAD attack, including previous angina pectoris with imaging examination indicating coronary atherosclerosis and/or lumen stenosis, whether acute or chronic [3]. The ethics review board of our hospital approved the study (ID:202102027), and the requirement for informed consent was waived owing to the retrospective design. The patients were excluded from the analysis for incomplete records after surgery; non-total arch or non-LCA surgery; and acute stage of stroke or neurological impairment (delirium, coma, psychosis, history of drug or alcohol abuse) at admission (Figure 1).

### 2.1. Operative Details

**Figure 2:**
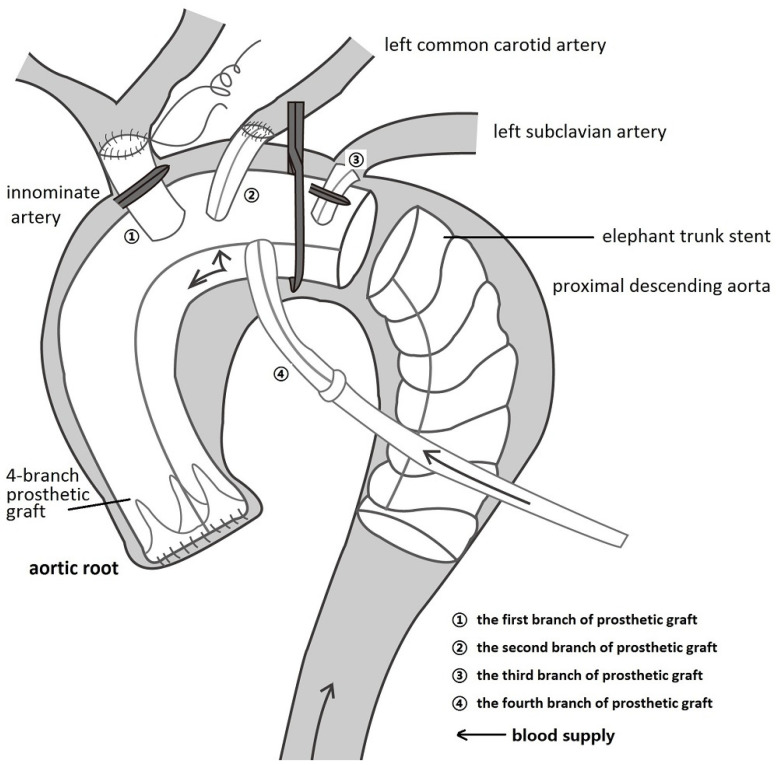
Myocardial priority diagram. The ascending aorta was replaced using the trunk of a 4-branched prosthetic graft (4BG), followed by anastomosis between the trunk and the aortic root, the left common carotid artery, and the second branch of the 4BG. The first branch and distal trunk of the 4BG were clipped, and the fourth branch of the 4BG was perfused by the Y-shaped bypass artery cannula, indicating that the blood supply to the coronary artery and left common carotid artery had recovered.

All patients were cared for by the same team of anesthesiologists, perfusionists, and intensivists but by different surgeons. Routine intraoperative monitoring is performed using a combination of transesophageal echocardiography (TEE), near-infrared spectroscopy (NIRS), electroencephalogram, electrocardiogram, and left/right radial and/or femoral artery pressure lines. After median sternotomy, cardiopulmonary bypass (CPB [11]) was performed through right atrial to femoral artery cannulation (with a Y-shaped artery cannula). During systemic cooling (25~27 °C) with a flow rate of 2.2~2.6 L/m^2^·min^−1^, antegrade cold cardioplegia was the preferred myocardial protection method. The ascending aorta was replaced with a 4-branched graft (4BG; Boston Scientific Inc., Boston, MA, USA) (Figure 2). If necessary, sinus plasty, valve replacement, or coronary reconstruction was performed. Subsequently, cardioplegia via the trunk of the 4BG was reperfused to check for aortic root anastomotic leakage.

**Figure 3:**
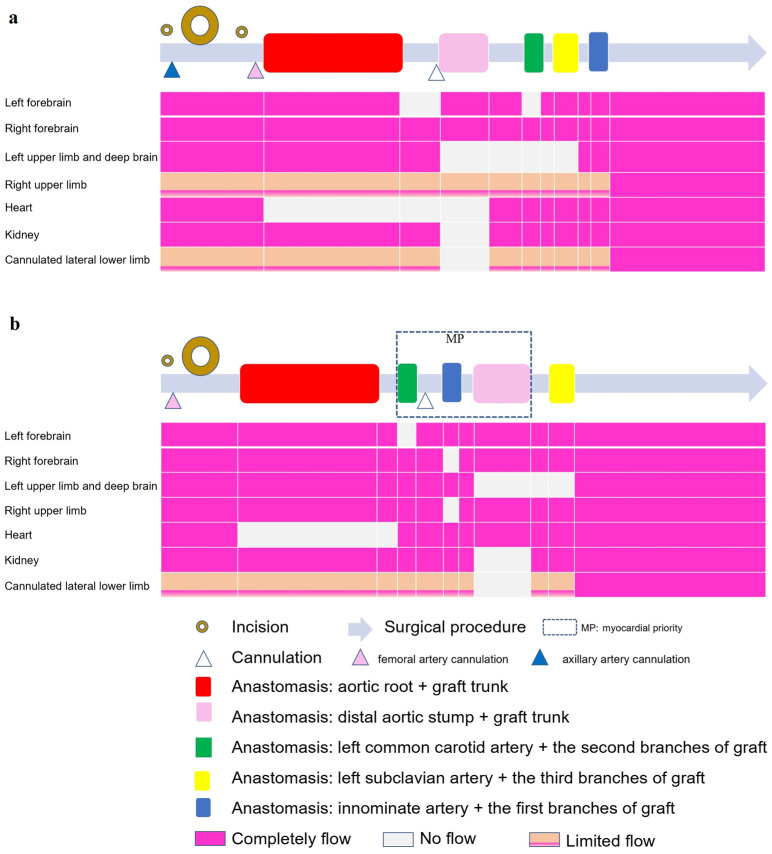
Illustration of surgical procedures (gray long arrow) in the two groups. (**a**) selective cerebral perfusion (SCP) group; (**b**) myocardial priority (MP) group. The hollow circles represent incisions, whereas the larger circles correspond to larger incisions. Triangles of different colors represent cannulations at different locations. The colored rectangles on the gray long arrow represent important anastomoses, corresponding to the perfusion flow of the following important organs. It was demonstrated that one incision and one cannulation were fewer in the MP group than in the other group. The blood flow of several organs illustrated that the forebrains of both groups were not completely ischemic; however, the ischemic time of the left deep brain and heart was shorter in the MP group, and the blood flow of the right upper limb was also less affected in the MP group.

In the traditional SCP group (Figure 3a), when the femoral artery cannula was clamped and LCA was instituted, a flow of 8~10 mL/min·kg^−1^ supplied the brain through the two cannulas (right axillary and left common carotid artery) [12]. A 12 cm CRONUS stented graft (SG; Microport Scientific Corp., Shanghai, China) was inserted into the true lumen of the descending aorta. Declamping of the CPB artery cannula and deairing of the grafts resulted in lower-body recovered perfusion. The left common carotid, left subclavian, and innominate arteries were anastomosed to the second, third, and first branches of the 4BG, respectively. The entire body is rewarmed during these steps.

In the MP group (Figure 3b), a Number-10 line was fastened between the third and fourth branches (Supplement Video S1) with a small catheter to close the trunk of the 4BG. The left common carotid artery was anastomosed to the second branch of the 4BG. The first branch of the 4BG was clipped, and the fourth branch of the 4BG was perfused by a Y-shaped CPB artery cannula, indicating that the blood supply to the coronary artery and left common carotid artery had recovered. Anastomosis of the innominate artery with the first branch of the 4BG was then performed. Next, the CPB femoral cannula was clamped and the LCA was instituted, with a flow of 15~20 mL/min·kg^−1^ to supply the fourth branch (bilateral cerebral hemisphere and heart, Figure 2). The SG was inserted into the descending aorta and anastomosed to the distal 4BG. After the lower body recovered perfusion, anastomosis of the left subclavian artery and third branch of the 4BG was performed while the whole body was rewarmed.

### 2.2. Data Collection

Demographic characteristics, comorbidities, type of surgery, TIMI Risk Score [13], intraoperative variables, data from perioperative laboratory tests, echocardiography results, duration of postoperative mechanical ventilation (MV), length of ICU stay and posthospital stay, and short-term outcomes (postoperative inotropic administration, postoperative bleeding score [14], transfusion, postoperative in-hospital morbidity and mortality) were recorded. Postoperative morbidity included low cardiac output, awake delay, temporary neurological dysfunction (TND), permanent neurological dysfunction (PND), stroke, spinal injury, re-exploration, adverse cardiac or cerebrovascular events (ACCE), pleural effusion/pneumothorax, acute lung injury, acute kidney injury (AKI), renal replacement therapy, acute hepatic injury, gastrointestinal injury, infection, and delayed incision healing (the definition of morbidities is shown in Supplement S1).

### 2.3. Outcome Measures

The primary outcome measure was posthospital stay, which was defined as the number of postoperative nights in the ICU and ward. The discharge criteria are presented in Supplement S1.

The secondary outcome measures included MV duration, ICU duration, ACCE and other in-hospital morbidities, mortality, monitoring, echocardiography and laboratory data, inotropic use, bleeding score, and transfusions.

Follow-up started from the day of hospital discharge and continued until the end of the study period (31 January 2021). Patients were contacted via telephone to collect data regarding clinic visits after discharge. Deaths and reasons for clinic visits were also recorded.

### 2.4. Analysis

Categorical variables were summarized as frequencies and percentages, while continuous variables were expressed as the mean ± standard deviation (SD) when the data were normally distributed and as the interquartile range (IQR) [P_50_(P_25_, P_75_)] when the data were nonnormally distributed. To generate two evenly matched cohorts of patients who received MP or SCP, we propensity score matched the patients using the following pre- and intraoperative variables: sex, age, weight, TIMI Risk Score, preoperative antiplatelet medicine, operation type, preoperative cerebral disease, NIRS base value < 50, cardiac surgery history, preoperative cTnI, preoperative CK-MB, preoperative EF, preoperative renal function, and minimum temperature. Of the 80 patients who received MP, we matched 79 MP patients with 79 patients who received SCP and underwent total aortic arch surgery. The matching tolerance was 0.1, and the propensity scores ranged from 0.14073 to 0.84112. The discrimination and calibration of propensity scores were assessed using the Hosmer–Lemeshow test and C-statistics, respectively. 

Continuous variables were compared with the use of two independent sample *t*-tests in matched pairs. Likewise, categorical variables were compared using the chi-square test or Fisher’s exact test, as appropriate. Laboratory variables over time were analyzed using a mixed-linear model for repeated measures, including laboratory factors for group, time, and the interaction between group and time. The variance–covariance matrix in the linear mixed-effects model was assumed to be unstructured. 

This trial was designed to investigate the potential superiority of MP in terms of shortened posthospital stays before statistical analysis. According to previous studies [11,15,16], we calculated that 56 patients per group would be sufficient, with a power of 0.8 and an alpha risk of 0.05. A *p* value < 0.05 was considered significant. The missing data were filled with multiple imputations. More than 20% of the CK-MB level data on postoperative Day 7 were missing; therefore, this parameter was removed. Data analysis was performed using IBM SPSS 23.0 (SPSS Software, IBM Corp., Armonk, NY, USA).

## 3. Results

### 3.1. Perioperative Characteristics and Outcomes

**Figure 4:**
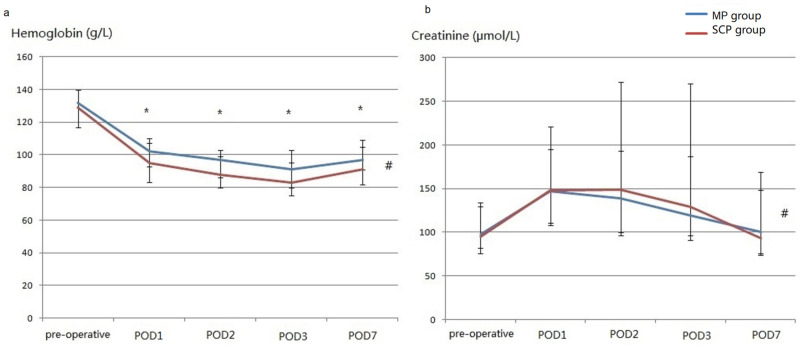
The perioperative concentration of hemoglobin (**a**) and creatinine (**b**) (after matching). (**a**), perioperative concentration of hemoglobin; (**b**), perioperative concentration of creatinine; POD, postoperative Day. # There were significant differences between the two groups. * Significant differences were observed between the two groups on the same postoperative day.

The patient demographics and baseline characteristics are shown in Table 1. The intraoperative variables, postoperative short-term outcomes, and perioperative laboratory data are shown in Table 2 and Table 3 and Figure 4, respectively. After propensity score matching, the demographic and preoperative variables of the two groups were similar. 

Compared with the SCP group, the MP group had a significantly higher perfusion flow and left radial pressure, a lower incidence of NIRS decrease of >20% of the base value, and a shorter myocardial ischemic time but longer LCA and CPB times. 

Furthermore, the MP group had a significantly shorter posthospital stay, smaller bleeding score and transfusions, and higher concentration of postoperative hemoglobin (*p* values of the between-group and interaction effects were 0.002 and 0.237, respectively). The postoperative creatinine levels in the MP group were also lower than those in the SCP group (*p* 0.041 and 0.167, respectively). Although between-group effects were insignificant for cTnI, CK-MB, platelets, total bilirubin, and PaO_2_/FiO_2_, the interactions of group with time were also significant for platelets, total bilirubin, and PaO_2_/FiO_2_ (Supplementary Figure S1). After removing interactions, there was no difference in the platelet count between the groups, and a higher bilirubin level on postoperative Day 3 and a higher PaO_2_/FiO_2_ on postoperative Day 7 were observed in the MP group (Supplement S2).

### 3.2. Subgroup Analysis

**Figure 5:**
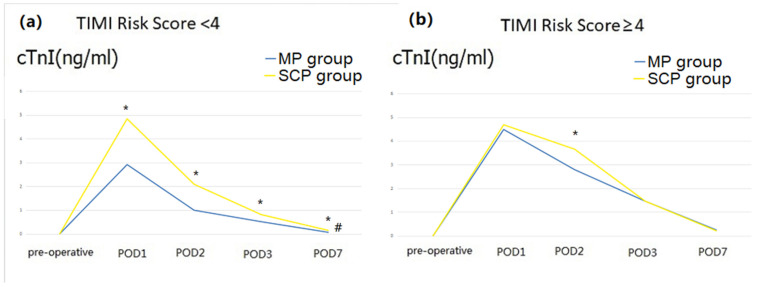
Perioperative cardiac troponin I (cTnI) level (after matching). (**a**), perioperative cTnI level when TIMA risk score < 4; (**b**), perioperative cTnI level when TIMA risk score ≥ 4; POD, postoperative Day. # There were significant differences between the two groups. * Significant differences were observed between the two groups on the same postoperative day.

We found a significant interaction between the group and the TIMI Risk Score (*p* = 0.022) that may affect posthospital stay (Supplement S3). After differentiating the TIMI Risk Score into two layers, the outcomes showed that when the TIMI Risk Score < 4, the MP group had a lower postoperative cTnI level (Figure 5) and lower incidence of low cardiac output (Table 4).

### 3.3. Follow-Up

The follow-up period ranged from 6 *to* 36 *months* (mean 20 months). No lost or new deaths occurred after hospital discharge. As shown in Supplement S4, 43 patients visited the clinic for various reasons (16/79 in the MP group vs. 27/79 in the SCP group), and most had a stroke (10 in the MP group vs. 11 in the SCP group).

## 4. Discussion

The main contribution of this study was that ATAAD patients complicated with CAD could benefit from a shorter posthospital stay and less blood loss and transfusion by the MP strategy possibly due to its shortened myocardial ischemia time and better maintained perfusion of vital organs, simplified surgical procedures, and altered anastomosis sequence. 

The MP strategy ensures bilateral physiological antegrade cerebral perfusion and simultaneous coronary blood flow. Although the CPB time increased slightly, it significantly reduced the posthospital stay (the median of the MP and SCP groups was 12 and 16 days, respectively). After removing early postoperative death cases that could also shorten the posthospital stay, the results remained the same. We then adjusted for some intraoperative factors that may affect the posthospital stay (Supplement S3), and the results of the MP group were still better than those of the SCP group, indicating that MP could enhance postoperative recovery. Another similar study introducing the “brain-heart-first” strategy for ATAAD raised the temperature to a mild hypothermia level of 30 °C, but the median hospital stay was still 16 days [17]. Chen et al.’s research reported that longer myocardial ischemia time and thrombocytopenia could predict longer posthospital stay in ATAAD surgery [18], the results of which were similar to ours.

The better vascular protection effects of less bleeding, transfusions, and higher postoperative hemoglobin in the MP group was also the probable reason for shortening the posthospital stay. It was similar to pediatric arch surgery, which could reduce chest tube time and platelet transfusion [19]. Although there are several factors (such as surgical skills, transfusion indications, priming or dilution of CPB circuits, and methods of lost blood collection [11]) that influence blood loss and transfusion, we thought that the main reason should be incision and cannulation reduction in the MP group, which is consistent with Lyu’s research that a minimally invasive approach could decrease transfusion in patients with type A dissection [20]. 

From the 1970s to the present, the proportion of ATAAD surgery combined with CAD has been high (20~30%) [21], while that combined with CABG is not high (5.6~7.5%) [22], and many articles indicate that CABG increases mortality [23,24]. Therefore, exploring new surgical strategies to improve the prognosis of ATAAD combined with CAD is clinically significant. Although the two groups had similar rates of return to spontaneous rhythm, postoperative cTnI and CK-MB levels, inotropic usage, incidence of low cardiac output, and postoperative EF values in our study, subgroup analysis showed that the postoperative cTnI level and incidence of low cardiac output decreased in the MP group when the TIMI Risk Score was <4. The higher the TIMI Score, the higher the risk of cardiac ischemic events in patients [13]. This means that minimizing the time of myocardial ischemia can reduce postoperative myocardial damage in ATAAD patients with non-severe CAD. This was consistent with previous studies that evaluated continuous myocardial perfusion during complex aortic arch repair [10,25]. Another important intraoperative factor influencing the myocardial protection effect is decompression [26]. Therefore, all hearts in our cases were empty during LCA (Supplementary Video S1), regardless of whether coronary blood supply recovery occurred. 

Moreover, the MP group had a higher radial pressure, and a lower incidence of NIRS decrease >20% of the base value, indicating better neuroprotective effects, although it had not yet reached the level where clinical outcomes such as postoperative awake delay, PND, and TND were significantly effective. This was consistent with some reports using the cerebral myocardial perfusion strategy; higher flow and pressure result in fewer complications [9,27]. ‘Branch first’ total arch replacement technique made left carotid-left subclavian bypass as first anastomosis, which also brings better neuroprotective effects [28]. Our modified MP strategy (Figure 3) might have some potential advantages except for changing the anastomotic sequence: the right radial MAP monitoring was direct and accurate during LCA, while stump pressures were in another group. The ischemic duration of the left deep brain (which could not be monitored by NIRS) might be shorter. No axillary cannulation affected blood flow in the right upper limb. 

No new deaths occurred during the 3 years of follow-up. Stroke and dissection were the most common complications in revisiting doctors. There was no significant difference in incidence of the total complications between the two groups. Among the 21 cases of stroke, four cases were not found during hospitalization (possibly new onset). MP was not associated with postoperative long-term morbidity reduction. The improvement in surgical strategy reduces morbidity insignificantly because of the mechanism of a high rate of distal aneurysmal evolution after type A aortic dissection repairment [29]. In a study of total arch surgery including dissection and aneurysm, malperfusion syndrome, CABG, tracheostomy, and AKI were associated with postoperative stroke, but the “beating heart” strategy (similar to MP) was not [10]. All patients in our cohort had type A dissections with a higher risk. After detecting these related factors (Supplement S5), we found that tracheostomy and intraoperative NIRS decreases of >20% of the baseline value were associated with postoperative stroke. 

This study had several limitations. As with all quality improvement studies, in comparison to randomized trials, our study aims to determine statistically significant associations between the intervention and the outcome, but cannot determine causation. Importantly, minor differences in surgical operations between surgeons were difficult to avoid; thus, the conclusions may have a certain degree of bias. Moreover, all data were retrieved retrospectively with a limited sample size from a single center and involved the small body size and low blood volume of Asian patients, and multiple tests including many outcomes in this small sample analysis; the 5% significance of the two parameters may be just a chance event [30]. These factors may limit the external validity of our findings. However, the data in this study were extracted from an electronic case record system, representing a relatively homogeneous cohort of patients, and the propensity score matching method reduced bias to some extent. Additionally, data were collected in a consistent manner using consistent definitions and practices.

## 5. Conclusions

Compared with the traditional strategy, the MP strategy for ATAAD patients with CAD showed simplified surgical procedures, protected the cardiovascular system, and promoted postoperative recovery, which is worthy of further study.

## Figures and Tables

**Table 1:** Patient characteristics before and after propensity score matching.

	MP (*n* = 80)	SCP (*n* = 134)	*p*	MP (*n* = 79)	SCP (*n* = 79)	*p*
Male sex (%)	65 (81%)	104 (78%)	0.527	64 (81%)	63 (80%)	0.841
Age (years)	51.00 (44.00, 58.75)	51.00 (42.75, 57.00)	0.266	51.00 (44.00, 59.00)	51.00 (42.00, 56.00)	0.325
Weight (kg)	71.00 (65.00, 80.00)	70.00 (60.00, 80.00)	0.171	72.00 (65.00, 80.00)	71.00 (64.00, 80.00)	0.921
TIMI Risk Score	2 (1, 4)	2 (1, 4)	0.336	2 (1, 4)	2 (1, 3)	0.116
Myocardial infarction within 90 days	1 (2%)	3 (4%)	0.605	1 (2%)	2 (3%)	0.560
Antiplatelet therapy before admission	31 (39%)	52 (39%)	0.994	29 (37%)	33 (42%)	0.515
Preoperative cerebral disease (%)	12 (15%)	19 (14%)	0.869	12 (15%)	9 (11%)	0.482
Cardiac surgery history (%)	7 (9%)	8 (6%)	0.441	7 (9%)	5 (%)	0.548
Preoperative cTnI (ng/mL)	0.01 (0, 0.03)	0.01 (0.01, 0.03)	0.02	0.01 (0.00, 0.03)	0.01 (0.005, 0.06)	0.055
Preoperative CK-MB (μ/L)	13.75 (9.83, 20.23)	12.65 (8.93, 18.70)	0.225	13.50 (9.80, 20.00)	12.70 (9.00, 19.00)	0.456
Preoperative EF (%)	60.00 (56.00, 65.00)	60.00 (56.00, 65.00)	0.765	60.00 (56.00, 65.00)	60.00 (56.00, 65.73)	0.939
NYHA grade (4/3/2 grade) (%)	9 (11%)	13 (10%)	0.736	9 (11%)	8 (10%)	0.967
	12 (15%)	16 (12%)		11 (14%)	11 (14%)	
	59 (74%)	105 (78%)		59 (75%)	60 (76%)	
Preoperative renal function (%) (Severe grade: on dialysis; severely impaired CC * < 50 mL/min off dialysis; moderately impaired CC were 50–85 mL/min; normal CC > 85 mL/min)	2 (3%)	4 (3%)	0.706	2 (3%)	3 (4%)	0.975
	19 (24%)	23 (17%)		18 (23%)	18 (23%)	
	39 (49%)	70 (52%)		39 (49%)	38 (48%)	
	20 (25%)	37 (28%)		20 (25%)	20 (25%)	
Hypertension (%)	63 (79%)	93 (69%)	0.137	62 (78%)	60 (76%)	0.704
COPD (%)	7 (9%)	6 (4%)	0.206	6 (8%)	4 (5%)	0.513

MP, myocardial priority; SCP, selective cerebral perfusion; TIMI, thrombolysis in Myocardial Infarction; cTnI, cardiac troponin I; CK-MB, creatine kinase myocardial isoenzyme; EF, ejection fraction; NYHA, New York Heart Association; COPD, chronic obstructive pulmonary disease; CC, creatinine clearance. * Creatinine clearance (ml/min) = (140-age (years)) × weight (kg) × (0.85 if female)/[72 × serum creatinine (mg/dL)], which was calculated using the Cochroft-Gault creatinine clearance calculator on the web (http://www.euroscore.org/calc.html, accessed on 20 August 2023).

**Table 2:** Intraoperative variables before and after propensity score matching.

	MP (*n* = 80)	SCP (*n* = 134)	*p*	MP (*n* = 79)	SCP (*n* = 79)	*p*
Type **Ⅰ** operation (%)	50 (62.5%)	80 (60%)	0.656	50 (63%)	50 (63%)	0.864
Type **Ⅱ** operation (%)	20 (25%)	31 (23.1%)		19 (24%)	17 (21.5%)	
Type **Ⅲ** operation (%)	10 (12.5%)	23 (17%)		10 (13%)	12 (15%)	
Perfusion flow when LCA (ml/kg min)	20.00 (15.00, 20.00)	10.00 (10.00, 10.00)	0.000 #	20.00 (15.00, 20.00)	10.00 (10.00, 10.00)	0.000 #
Left radial artery pressure	75 (70, 79.5)	73 (67.25, 76)	0.119	75 (70, 79)	71 (67, 76)	0.014
Temperature When LCA	25.00 (25.00, 25.68)	25.00 (24.49, 26.00)	0.283	25.00 (25.00, 25.70)	25.00 (24.60, 26.00)	0.863
Minimum Temperature	25.00 (24.33, 25.00)	24.65 (23.65, 25.20)	0.128	25.00 (24.40, 25.00)	25.00 (24.00, 25.20)	0.588
Base NIRS value < 50 (%)	8 (10%)	6 (4%)	0.114	8 (10%)	6 (8%)	0.576
Intraoperative NIRS value <50 (%)	9 (11%)	12 (9%)	0.585	9 (11%)	10 (13%)	0.807
Intraoperative NIRS decrease >20% base value (%)	5 (6%)	52 (39%)	0.000	5 (6%)	30 (38%)	0.000
LCA time (min)	29.00 (24.25, 36.00)	25.00 (19.00, 29.25)	0.000 #	29.00 (25.00, 36.00)	26.00 (21.00, 32.00)	0.003 #
CPB time (min)	224.50 (193.75, 264.25)	186.50 (166.00, 214.00)	0.000 #	225.00 (193.00, 265.00)	189.00 (165.00, 222.00)	0.000 #
Myocardial ischemic time (min)	79.00 (63.00, 99.25)	100.5 (78.75, 123.25)	0.000	79.00 (63.00, 100.00)	101.00 (79.00, 123.00)	0.000
Return to spontaneous rhythm (%)	18 (22.5%)	46 (34%)	0.067	18 (23%)	25 (32%)	0.211
Maximum Glucose (mmol/L)	12.70 (10.83, 14.30)	11.5 (10.18, 13.33)	0.028	12.70 (10.80, 14.30)	11.70 (10.40, 13.90)	0.202
Maximum Lactate (mmol/L)	5.50 (4.14, 7.30)	4.80 (3.50, 6.15)	0.052	5.50 (4.10, 7.30)	4.90 (3.65, 6.25)	0.155
Nadir Hb during CPB (g/dl)	6.65 (5.50, 7.10)	5.80 (5.10, 6.80)	0.002 #	6.80 (5.40, 7.10)	5.80 (5.10, 6.80)	0.005 #

MP, myocardial priority; SCP,: selective cerebral perfusion; Type **Ⅰ**, refers to ascending aortic replacement, total arch replacement, and proximal descending aortic stenting with elephant trunk; Type **Ⅱ**, refers to **Ⅰ** plus the proximal part of heart procedures: coronary artery bypass graft, and/or valve repair or replacement; Type **Ⅲ**, refers to **Ⅰ** plus distal portions of the aortic arch procedures: redo stenting, prior stent removal, complicated with spinal deformity, pregnancy, or cesarean; LCA: lower limb circulation arrest; NIRS, near-infrared spectroscopy; CPB, cardiopulmonary bypass; Hb, hemoglobin. # Larger value belonged to the MP group.

**Table 3:** Postoperative outcomes before and after propensity score matching.

		MP (*n* = 80)	SCP (*n* = 134)	*p*	MP (*n* = 79)	SCP (*n* = 79)	*p*
ICU stay (hours)		131.50 (89.75, 185.75)	128.50 (85.75, 187.25)	0.749	130.00 (89.00, 185.00)	140.00 (96.00, 205.00)	0.369
MV time (hours)		38.00 (25.00, 75.75)	44.00 (23.75, 77.50)	0.819	38.00 (25.00, 75.00)	52.00 (26.00, 85.00)	0.202
Posthospital stay (day) *		12.00 (8.25, 16.00)	15.00 (11.00, 21.00)	0.002	12.00 (8.00, 16.00)	16.00 (11.00, 21.00)	0.001
Bleeding Score		2.00 (2.00, 3.00)	2.00 (2.00, 3.00)	0.018	2.00 (2.00, 3.00)	2.00 (2.00, 3.00)	0.046
RBC transfusion (u)		4.00 (2.00, 7.50)	6.00 (3.50, 10.00)	0.005	4.00 (2.00, 7.50)	6.00 (3.50, 11.00)	0.018
Plasma transfusion (u)		8.60 (4.28, 14.38)	10.00 (6.00, 16.00)	0.094	8.30 (4.20, 14.00)	11.00 (6.00, 17.00)	0.093
Platelet transfusion (u)		1.00 (0.00, 1.00)	1.00 (1.00, 2.00)	0.000	1.00 (0.00, 1.00)	1.00 (1.00, 2.00)	0.000
Cryoprecipitate transfusion (u)		17.00 (0.00, 20.00)	19.50 (17.00, 35.63)	0.000	17.00 (0.00, 20.00)	19.50 (17.00, 36.00)	0.000
Inotropics	None (%)	11 (14%)	9 (7%)	0.194	11 (14%)	9(11%)	0.852
	Dop and/or NE (%)	44 (55%)	85 (63%)		44 (56%)	47(59.5%)	
	Dop and/or NE + E (%)	25 (31%)	40 (30%)		24 (30%)	23(29%)	
Low cardiac output (%)		17 (21%)	26 (19%)	0.744	16 (20%)	16 (20%)	1.000
Postoperative EF (%)		56.50 (51.00, 62.75)	58.00 (53.00, 63.00)	0.329	57.00 (51.00, 63.00)	58.00 (52.00, 63.00)	0.783
Awake delay (%)		27 (34%)	55 (41%)	0.288	26 (33%)	34 (43%)	0.190
TND (%)		29 (36%)	40 (30%)		29 (37%)	29 (37%)	1.000
PND (%)		13 (16%)	21 (16%)		12 (15%)	15 (18%)	0.526
Spinal injury (%)		3 (4%)	5 (4%)	0.994	3 (4%)	3 (4%)	1.000
ACCE (%)		29 (36.3%)	44 (32.8%)	0.610	28 (35.4%)	28 (35.4%)	1.000
Acute lung injury (%)		26 (32.5%)	54 (40%)	0.254	25 (32%)	33 (42%)	0.187
Pleural Space Drainage (%)		32 (40%)	47 (35%)	0.470	32 (40.5%)	32 (40.5%)	1.000
Post AKI (any grade) (%)		42 (52.5%)	81 (60%)	0.255	42 (53%)	52 (66%)	0.105
Post CRRT or renal failure (%)		6 (7.5%)	11 (8%)	0.853	5 (6%)	6 (8%)	0.755
Post ALI (%)		21 (26%)	39 (29%)	0.653	21 (27%)	22 (28%)	0.858
Post GI (%)		4 (5%)	3 (2%)	0.272	4 (5%)	2 (2.5%)	0.405
Incision delayed healing (%)		4 (5%)	7 (5%)	0.943	4 (5%)	6 (8%)	0.513
Infection (blood culture positive) (%)		10 (12.5%)	13 (10%)	0.522	10 (13%)	9 (11%)	0.807
Mortality (%)		5 (6%)	11 (8%)	0.598	4 (5%)	7 (9%)	0.348

MP, myocardial priority; SCP, selective cerebral perfusion; ICU, intensive care unit; MV, mechanical ventilation; RBC, red blood cells; Dop, dopamine; NE, norepinephrine; E, epinephrine; EF, ejection fraction; TND, temporary neurological dysfunction; PND, permanent neurological dysfunction; ACCE, adverse cardiac or cerebrovascular events; AKI, acute kidney injury; CRRT, continuous renal replacement treatment; ALI, acute liver injury; GI, gastriointestinal injury. * if short-term postoperative death occurs, which will also reduce the posthospital stay. The posthospital stay of the MP group was still shorter regardless of death cases (MP had 4 deaths and SCP had 7).

**Table 4:** Subgroup analysis according to whether TIMA risk score ≥4.

Outcomes	TIMI Risk Score < 4			TIMI Risk Score ≥ 4		
	MP (*n* = 43)	SCP (*n* = 53)	*p*	MP (*n* = 36)	SCP (*n* = 26)	*p*
Posthospital stay (day)	12 (8, 16)	15 (10, 19.5)	0.015	11.5 (9.3, 17)	17 (13, 23)	0.016
Low cardiac output (%)	1 (2.3%)	8 (15.1%)	0.033	15 (41.7%)	8 (30.8%)	0.381
Post AKI (any grade) (%)	22 (51.2%)	31 (58.5%)	0.473	20 (55.6%)	21 (80.8%)	0.038
Bleeding Score	2 (1, 2)	2 (2, 3)	0.043	2 (2, 3)	3 (2, 3)	0.260
RBC transfusion (u)	3 (1, 6)	4 (2.5, 9.8)	0.027	4.8 (2.3, 9.4)	7 (4.5, 15)	0.113
Platelet transfusion (u)	0 (0, 1)	1 (1, 2)	0.000	1 (0, 1.8)	1 (1, 3)	0.010
Cryoprecipitate transfusion (u)	17 (0, 19.5)	20 (17, 36)	0.001	18 (0, 30)	17.7 (17, 37)	0.146

TIMI, thrombolysis in Myocardial Infarction; MP, myocardial priority; SCP, selective cerebral perfusion; AKI, acute kidney injury.

## Data Availability

Full data can be requested from corresponding author, 404059@csu.edu.cn.

## Supplementary Materials

The following supporting information can be downloaded at: https://www.mdpi.com/article/10.3390/jpm13091296/s1.
