# Supplementary material for: Myocardial Priority Promotes Cardiovascular Recovery for Acute Type A Aortic Dissection Combined with Coronary Artery Disease Undergoing Aortic Arch Surgery
Source: J Pers Med. 2023 Aug 25;13(9):1296. doi: 10.3390/jpm13091296 (PMC10532919; doi:10.3390/jpm13091296)
Supplement: Supplementary file 1 [file jpm-13-01296-s001.zip › Supplement S1 -S5.pdf]

Supplement S1

The definitions of variables and morbidities

Posthospital stay: the number of postoperative nights in the ICU and ward. The criteria for discharge included (1) stability of vital signs with removal of the monitoring device and recovered autonomic activity, (2) medications or treatment not needed for supporting organ functions or absence of worsening organ function, (3) new versus old imaging showing patency of the graft and stent and repair of the dissection, or (4) death or refusal of further treatment.

Intraoperative radial mean artery pressure (MAP) was automatically recorded every 5 minutes, the number of minutes spent within MAP<55 mmHg or MAP>100 mmHg was duration of hypotension/hypertension.

Inotropic administration was defined according to three grades: 0, none; 1, the use of dopamine and/or noradrenaline; and 2, the use of dopamine and/or noradrenaline plus adrenaline for at least 1 h during the first 24 h postoperatively.

The bleeding score was recorded according to the universal definition of perioperative bleeding in adult cardiac surgery[1]: 0, insignificant; 1, mild; 2, moderate; 3, severe; or 4, massive.

Postoperative low cardiac output: need a high dose of inotropics (dopamine >15 $\mu g kg^{-1} min^{-1}$/ adrenaline >0.1 $\mu g kg^{-1} min^{-1}$/ noradrenaline >0.1 $\mu g kg^{-1} min^{-1}$) to maintain a systolic blood pressure greater than 90 mmHg or the need for mechanical circulation support [extracorporeal membrane oxygenation (ECMO) or intra-aortic balloon pump (IABP)] after the operation[2].

Postoperative awake delay: recovery of consciousness >24 h after surgery [3].

Postoperative temporary neurological dysfunction (TND): a symptom complex of postoperative confusion, seizure, agitation, or transient delirium with no structural abnormality in the brain detectable by the usual imaging methods, and resolution of the symptoms occurred usually before hospital discharge[4,5].

Postoperative permanent neurological dysfunction(PND): The new occurrence of postoperative neurological dysfunction at the time of discharge from the hospital, whether focal injury (stroke) or global (coma), when the focal lesion was confirmed by means of computed tomographic scanning or magnetic resonance imaging of the brain[4,5].

Postoperative stroke: postoperative neurological dysfunction whether by bleeding or infarction, confirmed by means of computed tomographic scanning or magnetic resonance imaging of the brain.

Postoperative spinal cord injury: postoperative hemiplegia, paraplegia, or motor deficits at discharge with imaging evidence of spinal cord ischemia[6].

Postoperative laryngeal nerve injury: postoperative dysphonia, dysphagia, or aspiration at discharge underwent fiberoptic laryngoscopy to confirm[7].

Re-exploration: re-operation for bleeding are chest tube output greater than 20% blood volume within 12-h of surgery or objective signs of cardiac tamponade or surgical bleeding[8].

Postoperative limb ischemia: new-onset ischemic event of the arterial system of the upper or lower limbs[9].

Postoperative adverse cardiac or cerebrovascular events (ACCE): as defined by the occurrence of 1 or more of the following 5 components during the procedure through hospital discharge: low cardiac output, PND, spinal cord injury, bleeding score 4, and limb ischemia.

Postoperative pleural effusion/pneumothorax: moderate to large amount of pleural effusion, hemothorax, or pneumothorax requiring closed-chest drainage.

Postoperative acute lung injury: new-onset respiratory failure, new-onset pulmonary embolism/oedema/pneumonia/massive pleural effusion, or hypoxemia exceeding 24 hours.

Postoperative acute kidney injury: new-onset renal failure requiring renal replacement or basline creatinine elevated by more than 50%[10–12].

Postoperative renal replacement therapy: new onset of renal failure and requiring dialysis or CRRT (continuous renal replacement therapy).

Postoperative acute hepatic injury: new-onset hepatic failure or total bilirubin >51 μmol/L[13].

Postoperative gastrointestinal injury: new-onset stress ulcer, gastrointestinal haemorrhage or melena.

Postoperative infection: clinical signs of sepsis (anomalous body temperature, increased heart rate, increased respiratory rate) combined with a positive blood culture[14].

Delayed incision healing: The incision is red and swollen, purulent, secretive or bacterial culture positive, and still requiring replacement of accessories after discharge[15].

Malperfusion syndrome: preoperative focal neurological deficit ± myocardial infarction ± visceral ischaemia ± peripheral/limb ischaemia[16].

Ref:

1. Dyke, C.; Aronson, S.; Dietrich, W.; Hofmann, A.; Karkouti, K.; Levi, M.; Murphy, G.J.; Sellke, F.W.; Shore-Lesserson, L.; von Heymann, C.; et al. Universal Definition of Perioperative Bleeding in Adult Cardiac Surgery. *The Journal of Thoracic and Cardiovascular Surgery* **2014**, *147*, 1458-1463.e1, doi:10.1016/j.jtcvs.2013.10.070.
2. Sanetra, K.; Gerber, W.; Shrestha, R.; Domaradzki, W.; Krzych, Ł.; Zembala, M.; Cisowski, M. The Del Nido versus Cold Blood Cardioplegia in Aortic Valve Replacement: A Randomized Trial. *The Journal of Thoracic and Cardiovascular Surgery* **2019**, *159*, 2275–2283, doi:10.1016/j.jtcvs.2019.05.083.
3. Wang, Z.-Y.; Gu, W.-J.; Luo, X.; Ma, Z.-L. Risk Factors of Delayed Awakening after Aortic Arch Surgery under Deep Hypothermic Circulatory Arrest with Selective Antegrade Cerebral Perfusion. *J. Thorac. Dis* **2019**, *11*, 805–810, doi:10.21037/jtd.2019.02.01.
4. Liu, H.; Chang, Q.; Zhang, H.; Yu, C. Predictors of Adverse Outcome and Transient Neurological Dysfunction Following Aortic Arch Replacement in 626 Consecutive Patients in China. *Heart, Lung and Circulation* **2017**, *26*, 172–178, doi:10.1016/j.hlc.2016.02.004.
5. Gatti, G.; Benussi, B.; Currò, P.; Forti, G.; Rauber, E.; Minati, A.; Gabrielli, M.; Tognolli, U.; Sinagra, G.; Pappalardo, A. The Risk of Neurological Dysfunctions

after Deep Hypothermic Circulatory Arrest with Retrograde Cerebral Perfusion. *Journal of Stroke and Cerebrovascular Diseases* **2017**, *26*, 3009–3019, doi:10.1016/j.jstrokecerebrovasdis.2017.07.034.
6. Yoshitani, K.; Masui, K.; Kawaguchi, M.; Kawamata, M.; Kakinohana, M.; Kato, S.; Hasuwa, K.; Yamakage, M.; Yoshikawa, Y.; Nishiwaki, K.; et al. Clinical Utility of Intraoperative Motor-Evoked Potential Monitoring to Prevent Postoperative Spinal Cord Injury in Thoracic and Thoracoabdominal Aneurysm Repair: An Audit of the Japanese Association of Spinal Cord Protection in Aortic Surgery Database. *Anesthesia & Analgesia* **2018**, *126*, 763–768, doi:10.1213/ANE.0000000000002749.
7. Lodewyks, C.L.; White, C.W.; Bay, G.; Hiebert, B.; Wu, B.; Barker, M.; Kirkpatrick, I.; Arora, R.C.; Moon, M.; Pascoe, E. Vocal Cord Paralysis After Thoracic Aortic Surgery: Incidence and Impact on Clinical Outcomes. *The Annals of Thoracic Surgery* **2015**, *100*, 54–58, doi:10.1016/j.athoracsur.2015.02.021.
8. Ohmes, L.B.; Di Franco, A.; Guy, T.S.; Lau, C.; Munjal, M.; Debois, W.; Li, Z.; Krieger, K.H.; Schwann, A.N.; Leonard, J.R.; et al. Incidence, Risk Factors, and Prognostic Impact of Re-Exploration for Bleeding after Cardiac Surgery: A Retrospective Cohort Study. *International Journal of Surgery* **2017**, *48*, 166–173, doi:10.1016/j.ijsu.2017.10.073.
9. Folkert, I.W.; Foley, P.J.; Wang, G.J.; Jackson, B.M.; Bavaria, J.E.; Desai, N.D.; Fairman, R.M.; Damrauer, S.M. Impact of Acute Postoperative Limb Ischemia after Cardiac and Thoracic Aortic Surgery. *Journal of Vascular Surgery* **2018**, *67*, 1530-1536.e2, doi:10.1016/j.jvs.2017.09.019.
10. Hu, G.-H.; Duan, L.; Jiang, M.; Zhang, C.-L.; Duan, Y.-Y. Wider Intraoperative Glycemic Fluctuation Increases Risk of Acute Kidney Injury after Pediatric Cardiac Surgery. *Renal Failure* **2018**, *40*, 611–617, doi:10.1080/0886022X.2018.1532908.
11. Duan, L.; Hu, G.-H.; Jiang, M.; Zhang, C.-L.; Duan, Y.-Y. Association of Hypoalbuminemia with Acute Kidney Injury in Children after Cardiac Surgery. *Chin J Contemp Pediatr* **2018**, *20*, 475–480.
12. DUAN Lian, HU Guo-Huang, JIANG Meng, et al. Clinical Characteristics and Prognostic Analysis of Children with Congenital Heart Disease Complicated by Postoperative Acute Kidney Injury. *Chin J Contemp Pediatr* **2017**, *19*, 1196–1202, doi:10.7499/j.issn.1008-8830.2017.11.014.
13. Duan, L.; Hu, G.; Jiang, M.; Zhang, C. Postoperative abnormal liver function in children with heart surgery. *Zhong Nan Da Xue Xue Bao Yi Xue Ban* **2018**, *43*, 1007–1013, doi:10.11817/j.issn.1672-7347.2018.09.012.
14. van Koeverden, I.D.; den Ruijter, H.M.; Scholtes, V.P.W.; G. E. H. Lam, M.; Haitjema, S.; Buijsrogge, M.P.; J. L. Suyker, W.; van Wijk, R.H.; de Groot, M.C.H.; van Herwaarden, J.A.; et al. A Single Preoperative Blood Test Predicts Postoperative Sepsis and Pneumonia after Coronary Bypass or Open Aneurysm Surgery. *Eur J Clin Invest* **2019**, *49*, e13055, doi:10.1111/eci.13055.
15. Piwnica-Worms, W.; Azoury, S.C.; Kozak, G.; Nathan, S.; Stranix, J.T.; Colen, D.; Othman, S.; Vallabhajosyula, P.; Serletti, J.; Kovach, S. Flap Reconstruction for Deep Sternal Wound Infections: Factors Influencing Morbidity and Mortality. *The Annals of Thoracic Surgery* **2020**, *109*, 1584–1590, doi:10.1016/j.athoracsur.2019.12.014.
16. Martens, A.; Beckmann, E.; Kaufeld, T.; Umminger, J.; Fleissner, F.; Koigeldiyev, N.; Krueger, H.; Puntigam, J.; Haverich, A.; Shrestha, M. Total Aortic Arch Repair: Risk Factor Analysis and Follow-up in 199 Patients. *Eur J Cardiothorac Surg* **2016**, *50*, 940–948, doi:10.1093/ejcts/ezw158.

Supplement S2

Pairwise comparison of individual effects of grouping at a fixed time at a certain level

item	time	MP	non-CMP	MD	SE	*P*	95%CI	
Platelet count	POD1	1	0	-4.026	6.615	.544	-17.094	9.042
	POD2	1	0	-4.758	8.470	.575	-21.489	11.973
	POD3	1	0	4.556	9.469	.631	-14.149	23.262
	POD7	1	0	28.853	18.019	.111	-6.741	64.447
Bilirubin	POD1	1	0	-2.442	4.246	.566	-10.830	5.947
	POD2	1	0	6.722	3.792	.078	-.768	14.213
	POD3	1	0	10.975	4.225	.010	2.629	19.321
	POD7	1	0	8.181	5.386	.131	-2.459	18.821
PaO_2/FiO_2	POD1	1	0	-.148	.107	.170	-.360	.064
	POD2	1	0	-.077	.123	.531	-.320	.166
	POD3	1	0	-.052	.140	.709	-.329	.224
	POD7	1	0	-.413	.156	.009	-.721	-.106

MP, myocardial priority; POD, postoperative Day; PaO_2/FiO_2, arterial oxygen partial pressure to inhaled oxygen concentration.

Supplement S3

Exploring interaction effects between intraoperative variables and MP/SCP grouping that may affect posthospital stay (linear regression model)

Variables	Interaction *P*	Variables *P*	Group *P*	Model *P*
Surgeons(1/2/3/4/5)	0.128	0.133	0.269	0.025
Introperative NIRS value<50	0.792	0.884	0.095	0.041
Introperative NIRS decrease>20% base value	0.946	0.234	0.171	0.017
Grade* of duration of MAP<55mmHg	0.022	0.092	0.000	0.003
LCA time ≥30 min	0.851	0.359	0.009	0.028
CPB time ≥210 min	0.810	0.921	0.010	0.042
Cardiac ischemic time ≥90 min	0.868	0.185	0.000	0.019
Return to spontaneous rhythm	0.616	0.760	0.007	0.036

MP, myocardial priority; SCP, selective cerebral perfusion; NIRS, near infrared spectroscopy; MAP, mean artery pressure; LCA: lower limb circulation arrest; CPB, cardiopulmonary bypass.

*Grade: 1, duration of MAP<55mmHg less than 30 minutes; 2, 30 minutes≤ 'duration of MAP<55mmHg' ≤60 minutes, 3, duration of MAP<55mmHg more than 60 minutes

Supplement S4

Follow up data illustration

	Total number	MP(n=79)	SCP(n=79)
Stroke (n)	21(new onset 4)	10(new onset 2)	11(new onset 2)
Dissection (residual or new onset) (n)	8(new onset 1)	2	6(new onset 1)
Incision delayed healing (n)	3	0	3
Kidney dysfunction (n)	2	2	0
Lower or upper limb problems# (n)	3	1	2
Herpes progenitalis* (n)	1	1	0
Under/over anticoagulation (n)	2	1	1
Severe pericardial effusion (n)	1	0	1
Cardiac dysfunction (n)	1	0	1
Chest pain (n)	2	0	2
Total (n)	44	17	27

MP, myocardial priority; SCP, selective cerebral perfusion.

#refered to osteofascial compartment syndrome, varicosity, poor muscle strength (local image negative) each.

* the patient of herpes progenitalis also concurrent with kidney dysfunction.

The two groups had similar rates of total morbidites, stroke, dissection, and incision delayed healing (the *p* value were 0.076, 0.815, 0.147, 0.123, respectively)

Supplement S5

Risk factors analysis of postoperative stroke (during hospitalization and follow up) in matched patients

	Univariate logistic regression			Multivariate logistic regression		
	P	OR	95%CI	*P*	OR	95%CI
malperfusion syndrome	0.612	0.776	0.291-2.069			
CABG	0.709	1.211	0.443-3.315			
Tracheostomy	0.000	29.762	6.088-145.506	0.001	16.195	2.991-87.695
AKI	0.150	1.867	0.797-4.374			
MP	0.548	0.786	0.357-1.728			
Introperative NIRS decrease>20% base value	0.000	5.016	2.142-11.746	0.013	3.430	1.303-9.033
Grade* of duration of MAP<55mmHg	0.970	0.990	0.582-0.683			
Bleeding Score	0.001	2.150	1.352-3.420			
Surgeons(1/2/3/4/5)	0.240	1.181	0.895-1.559			

CABG, coronary artery bypass graft; AKI, acute kidney injury; MP, myocardial priority; NIRS, near infrared spectroscopy; MAP, mean artery pressure.

*Grade: 1, duration of MAP<55mmHg less than 30 minutes; 2, 30 minutes≤ 'duration of MAP<55mmHg' ≤60 minutes, 3, duration of MAP<55mmHg more than 60 minutes.

Supplementary Figure S1 Legend:

Figure S1: Perioperative concentration of cTnI, CK-MB, hemoglobin, platelet, creatinine, total bilirubin and PaO_2/FiO_2 (after matching)

MP: myocardial priority; cTnI, cardiac troponin I; CK-MB, creatine kinase myocardial isoenzyme; PaO_2/FiO_2: arterial oxygen partial pressure to inhaled oxygen concentration; POD: postoperative Day.

Pb: P value of between-group effect

Pi: P value of interaction effect

Supplementary Video S1: After stented graft implantation and before anastomosis of distal aorta, the heart is beating and empty during hypothermic lower body circulation arrest.
