# Supplementary figures and images for: Myocardial Priority Promotes Cardiovascular Recovery for Acute Type A Aortic Dissection Combined with Coronary Artery Disease Undergoing Aortic Arch Surgery
Source: J Pers Med. 2023 Aug 25;13(9):1296. doi: 10.3390/jpm13091296 (PMC10532919; doi:10.3390/jpm13091296)

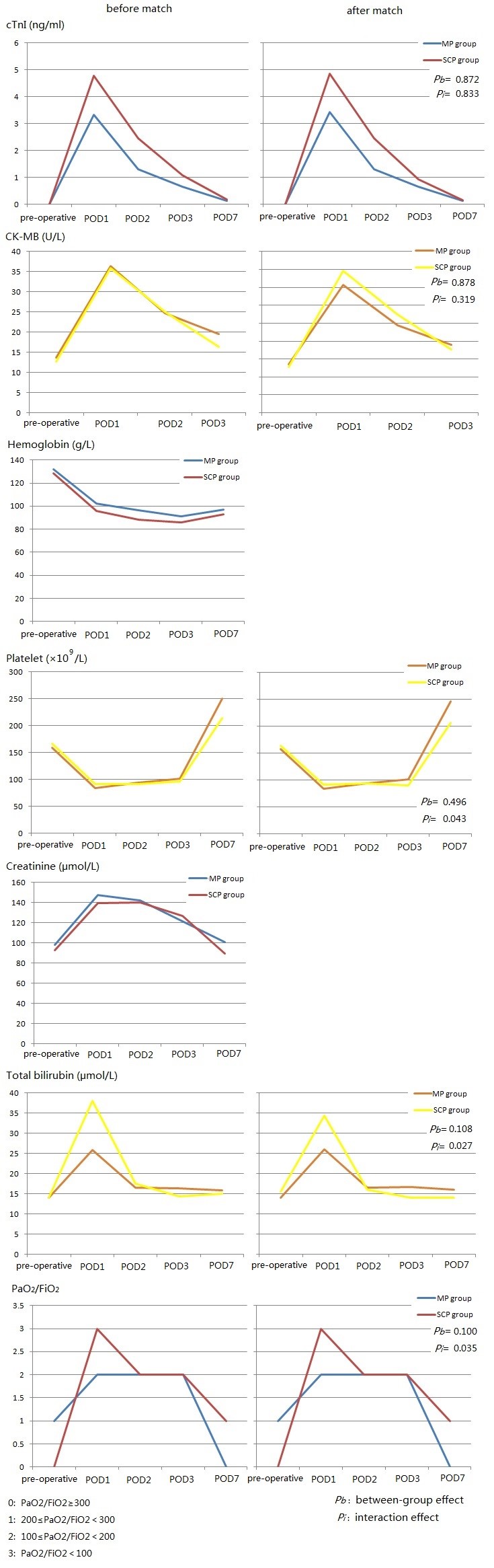

Supplement: Supplementary file 1 [file jpm-13-01296-s001.zip › sFig1 other lab data.jpg]
